# Integration of Electronic Invoices in Healthcare Finance: A Case Study of Ministry of Health Hospitals in Saudi Arabia

**DOI:** 10.7759/cureus.71712

**Published:** 2024-10-17

**Authors:** Abdullah A Alshameri, Eidhah M Alnahdi, Fahad A Bahamdain, Lafe O Almutairi, Omar A Alshaikhi, Omar Alamri

**Affiliations:** 1 Department of Medical Insurance Office, East Jeddah Hospital, Jeddah, SAU; 2 Department of Infection Control, King Abdul-Aziz University Hospital, Jeddah, SAU

**Keywords:** e-invoice system, electronic invoicing, healthcare finance, ministry of health hospitals, operational efficiency, physician training, saudi arabia, system integration, technical challenges, user satisfaction

## Abstract

Background: The integration of electronic invoicing (E-invoicing) systems in healthcare finance is becoming increasingly crucial for enhancing operational efficiency. Despite the potential benefits, the successful implementation of these systems often faces challenges related to physician training, technical issues, and system integration.

Objective: This study aims to explore the effectiveness of E-invoice systems in Ministry of Health Hospitals in Saudi Arabia, focusing on enhancing physician training, addressing technical challenges, and promoting system integration to improve overall healthcare service delivery.

Methods: A comprehensive approach was adopted, involving qualitative assessments of current E-invoice implementation practices, training needs analysis, and stakeholder feedback. Data were collected through surveys and interviews with healthcare professionals, administrators, and IT specialists to identify challenges and recommend solutions.

Results: The findings highlight the need for tailored training programs that encompass various aspects of the E-invoice system. Key technical challenges identified include system compatibility, user interface design, and connectivity issues. Integration of the E-invoice system with existing hospital workflows was shown to enhance data accuracy, streamline processes, and improve user satisfaction.

Conclusion: The successful implementation of the E-invoice system in healthcare settings hinges on comprehensive training, proactive technical support, and effective integration strategies. Addressing these areas will not only optimize billing and reimbursement processes but also foster a collaborative environment that enhances patient care and organizational sustainability.

## Introduction

The integration of electronic invoices (E-invoices) in healthcare finance is a crucial step toward modernizing healthcare systems, enhancing operational efficiency, and ensuring transparency in financial transactions. This study examines the implementation of electronic invoicing systems at Ministry of Health Hospitals in Saudi Arabia, exploring both the adoption process and its implications within the healthcare sector [1-4].

In recent years, Saudi Arabia has actively pursued digital transformation initiatives across various sectors, including healthcare. This commitment to digitalization aims to streamline processes, minimize errors, and enhance accountability in public services [2-5]. The implementation of electronic invoicing systems in healthcare aligns seamlessly with these goals, improving the efficiency of financial management practices. Insights from international experiences offer valuable lessons regarding the regulatory and policy frameworks essential for integrating electronic invoicing systems within financial services. The sensitive nature of healthcare finance and its regulatory environment underscore the importance of leveraging best practices from other contexts to inform implementation strategies in Saudi Arabia [5-9].

The adoption of hospital information systems has revealed critical factors that influence the acceptance and utilization of electronic invoicing systems in healthcare finance. Key considerations include performance expectancy, effort expectancy, social influence, and facilitating conditions, which all play a significant role in driving technology adoption [6-10]. Understanding these factors can help identify barriers and facilitators specific to the electronic invoicing landscape within Saudi Arabian healthcare settings.

The COVID-19 pandemic has further emphasized the need for agile and resilient financial systems. Electronic invoicing systems can significantly enhance the adaptability of healthcare finance by enabling remote processing, reducing physical contact, and ensuring continuity in financial operations during crises [7-11].

Integrating electronic invoicing within healthcare finance can yield several benefits for the Saudi Arabian context. It can streamline the invoicing process, reducing paperwork and administrative burdens on healthcare professionals. Moreover, it can enhance the accuracy and transparency of financial transactions, thereby mitigating risks associated with errors and fraud. Additionally, facilitating timely payments to healthcare providers can improve liquidity and operational efficiency within the healthcare system [5-10].

Nonetheless, the successful implementation of electronic invoicing systems in healthcare finance necessitates addressing several challenges, including ensuring interoperability with existing healthcare information systems, addressing data security and privacy concerns, providing adequate training and support to healthcare staff, and complying with regulatory standards. Therefore, the integration of electronic invoicing systems represents a significant opportunity for modernizing financial management practices in Saudi Arabia's healthcare system. By drawing on international experiences, leveraging theoretical frameworks, and proactively addressing implementation challenges, Saudi Arabia can unlock the full potential of electronic invoicing systems in transforming healthcare finance operations. This study also emphasizes the technical challenges reported in similar contexts and incorporates insights into the essential aspects for successful electronic invoice implementation.

## Materials and methods

Study design

This study employed a mixed-methods research design to explore the integration of electronic invoices (E-invoices) within the medical insurance system at Ministry of Health Hospitals in Saudi Arabia. The research aimed to achieve three primary objectives: identifying technical challenges, evaluating the specific needs of medical insurance physicians, and determining their expectations regarding the E-invoice system.

Data collection

Data were collected using a two-pronged approach. First, a comparative analysis was conducted to measure key performance indicators related to healthcare financial operations before and after the implementation of the E-invoice system. Metrics such as invoice processing references, error rates, and clearance efficiency were evaluated to assess the impact of electronic invoicing on the financial landscape of the healthcare sector.

Second, a structured questionnaire was distributed to 12 medical insurance physicians working within Ministry of Health facilities. This survey aimed to gauge their awareness, perceptions, and expectations of the E-invoice system. Key focus areas included familiarity with the E-invoice system, perceived advantages and disadvantages, and any challenges encountered during its use. A stratified random sampling approach was employed to ensure diverse representation among physicians, capturing a broad range of experiences and perspectives.

Data analysis

Quantitative data collected from the surveys were analyzed using descriptive statistics to calculate awareness levels and expectations among medical insurance physicians. Qualitative responses were synthesized to provide contextual insights into the challenges and perceptions related to E-invoice integration. The integration of both data sources yielded a comprehensive and objective assessment of the difficulties facing E-invoice adoption within Ministry of Health Hospitals.

Ethical considerations

Ethical considerations were critical in this research. The Jeddah First Health Cluster issued approval A01839. Measures were taken to protect the anonymity of participants and ensure informed consent. Medical insurance physicians were provided with a clear understanding of the study’s aims, methods, and potential risks before participating. Anonymity was maintained throughout the study, and all data were stored securely in accordance with ethical standards and regulations, prioritizing confidentiality and the autonomy of participants.

## Results

Frequency analysis

The study involved 99 medical insurance physicians from Ministry of Health Hospitals, providing valuable insights into their experiences with the E-invoice system. On average, respondents reported working as medical insurance physicians for approximately 1.31 years, with an average frequency of using the E-invoice system of 1.33 times per week. However, the overall experience with the system received a low satisfaction rating of 1.22 out of 5, indicating significant dissatisfaction and challenges faced by the physicians. Among the reported technical challenges, the average rating was 1.24 out of 5, highlighting issues such as system compatibility, user interface difficulties, connectivity problems, and software errors (Table 1).

**Table 1:** Experience and usage of the E-invoice system by medical insurance physicians "N Valid" represents the number of valid responses, while "N Missing" indicates the number of responses not recorded. "Mean Rating" is on a scale from 1 to 5, with higher values indicating a more positive response.

Question	N Valid	N Missing	Mean Rating
How long have you been working as a medical insurance physician at Ministry of Health Hospitals?	99	0	1.31
How often do you currently use the E-invoice system?	99	0	1.33
How would you rate your overall experience with the E-invoice system?	99	0	1.22
What technical challenges have you encountered while using the E-invoice system?	99	0	1.24

When queried about training adequacy, respondents rated their training experience at 1.19 out of 5, signifying that most felt that they had not received sufficient training on the E-invoice system. In terms of the system's impact on workflow efficiency, the average rating was 1.24, further suggesting that it had not significantly improved their workflow. Respondents expressed a desire for improvements, with an average rating of 2.76 out of 5, identifying areas such as faster processing times, enhanced security features, and a more user-friendly interface. The perceived importance of integrating electronic invoices for improving billing efficiency garnered an average rating of 1.49, while overall satisfaction with the E-invoice system was rated at 1.30 out of 5. Despite these challenges, many respondents indicated that they would recommend the system to their colleagues, reflected in an average rating of 1.19 (Table 2).

**Table 2:** Training, impact, and satisfaction with the E-invoice system "N Valid" represents the number of valid responses, while "N Missing" indicates the number of responses not recorded. "Mean Rating" is on a scale from 1 to 5, with higher values indicating a more positive response.

Question	N Valid	N Missing	Mean Rating
Have you received adequate training on how to use the E-invoice system?	99	0	1.19
How would you describe the impact of the E-invoice system on your workflow efficiency?	99	0	1.24
What improvements would you like to see in the E-invoice system?	99	0	2.76
How important is the integration of E-invoices in improving the efficiency of medical insurance billing processes at Ministry of Health Hospitals?	99	0	1.49
Overall, how satisfied are you with the E-invoice system?	99	0	1.30
Would you recommend the E-invoice system to other medical insurance physicians?	99	0	1.19

Correlation analysis

The correlation analysis revealed significant relationships between various variables regarding the use of the E-invoice system. A noteworthy positive correlation was found between the length of time working as a medical insurance physician and the technical challenges encountered while using the E-invoice system. This suggests that more experienced physicians may face distinct challenges compared to those with less experience. Furthermore, a positive correlation between adequate training and overall satisfaction indicated that proper training could enhance the user experience (Table 3).

**Table 3:** Correlations between E-invoice system usage and physician feedback Correlation values are Pearson correlation coefficients. Asterisks indicate significance levels: **p < 0.01 and p < 0.05. Sig. (2-tailed) values indicate the statistical significance of the correlations. N refers to the number of valid responses for each item.

Question	How long have you been working as a medical insurance physician at Ministry of Health Hospitals?	How often do you currently use the E-invoice system?	How would you rate your overall experience with the E-invoice system?	What technical challenges have you encountered while using the E-invoice system?	Have you received adequate training on how to use the E-invoice system?	How would you describe the impact of the E-invoice system on your workflow efficiency?	What improvements would you like to see in the E-invoice system?	How important is the integration of E-invoices in improving the efficiency of medical insurance billing processes at Ministry of Health Hospitals?	Overall, how satisfied are you with the E-invoice system?	Would you recommend the E-invoice system to other medical insurance physicians?
Pearson correlation	1	0.354**	0.190	0.381**	0.094	0.246*	-0.253*	0.151	0.365**	-0.046
Sig. (2-tailed)		0.000	0.059	0.000	0.354	0.014	0.012	0.135	0.000	0.648
How often do you currently use the E-invoice system?	0.354**	1	0.137	0.400**	0.136	0.250*	0.125	-0.012	0.038	0.091
Sig. (2-tailed)	0.000		0.175	0.000	0.178	0.013	0.216	0.906	0.706	0.372
How would you rate your overall experience with the E-invoice system?	0.190	0.137	1	0.151	-0.187	-0.189	0.008	-0.118	-0.029	0.356**
Sig. (2-tailed)	0.059	0.175		0.135	0.064	0.061	0.938	0.243	0.776	0.000
What technical challenges have you encountered while using the E-invoice system?	0.381**	0.400**	0.151	1	0.247*	0.285**	0.019	0.045	0.199*	0.083
Sig. (2-tailed)	0.000	0.000	0.135		0.014	0.004	0.853	0.661	0.048	0.412
Have you received adequate training on how to use the E-invoice system?	0.094	0.136	-0.187	0.247*	1	0.473**	-0.009	-0.016	0.356**	0.083
Sig. (2-tailed)	0.354	0.178	0.064	0.014		0.000	0.927	0.872	0.000	0.415
How would you describe the impact of the E-invoice system on your workflow efficiency?	0.246*	0.250*	-0.189	0.285**	0.473**	1	-0.050	-0.154	0.410**	-0.036
Sig. (2-tailed)	0.014	0.013	0.061	0.004	0.000		0.622	0.127	0.000	0.722
What improvements would you like to see in the E-invoice system?	-0.253*	0.125	0.008	0.019	-0.009	-0.050	1	0.031	-0.119	0.040
Sig. (2-tailed)	0.012	0.216	0.938	0.853	0.927	0.622		0.759	0.243	0.693
How important is the integration of E-invoices in improving the efficiency of medical insurance billing processes at Ministry of Health Hospitals?	0.151	-0.012	-0.118	0.045	-0.016	-0.154	0.031	1	0.096	-0.234*
Sig. (2-tailed)	0.135	0.906	0.243	0.661	0.872	0.127	0.759		0.344	0.020
Overall, how satisfied are you with the E-invoice system?	0.365**	0.038	-0.029	0.199*	0.356**	0.410**	-0.119	0.096	1	-0.035
Sig. (2-tailed)	0.000	0.706	0.776	0.048	0.000	0.000	0.243	0.344		0.733
Would you recommend the E-invoice system to other medical insurance physicians?	-0.046	0.091	0.356**	0.083	0.083	-0.036	0.040	-0.234*	-0.035	1
Sig. (2-tailed)	0.648	0.372	0.000	0.412	0.415	0.722	0.693	0.020	0.733	
N	99	99	99	99	99	99	99	99	99	99

The analysis also indicated a positive correlation between the perceived impact of the E-invoice system on workflow efficiency and overall satisfaction. Physicians who recognized a positive effect on their workflow were more likely to express satisfaction with the system. Conversely, a negative correlation was noted between desired improvements in the E-invoice system and overall satisfaction, suggesting that those who identified a need for enhancements were less satisfied with the current system.

## Discussion

Electronic invoicing facilitates faster payments and approval processes, enabling early payment options. This streamlined payment mechanism enhances supplier relationships, fostering customer loyalty and retention. E-invoicing benefits both buyers and suppliers by significantly reducing invoice processing costs and eliminating manual tasks [4-9]. Traditional paper invoices incur high operational costs, while E-invoicing automates the entire process, removing the need for physical documentation. This transition allows accounting teams to concentrate on strategic tasks, ultimately resulting in higher levels of customer satisfaction.

As a crucial aspect of modern business operations, E-invoicing offers numerous advantages, including cost savings, increased efficiency, automation, compliance, sustainability, and reduced human error. By streamlining the invoicing process, businesses can save over 60% in time and associated invoicing costs, thereby allowing greater focus on other critical areas of the business [10-15].

E-invoicing eliminates the reliance on paper and inefficient digital invoices, enabling organizations to optimize their processes and conserve both time and resources. It also reduces administrative costs linked to traditional invoicing methods, allowing for better allocation of resources to other operational areas. E-invoicing enhances efficiency by removing the need for manual data entry, printing, and mailing, thus automating the invoicing process, minimizing human error, and ensuring data accuracy. Furthermore, E-invoicing aids compliance with tax legislation, facilitating business operations across different countries [6-9].

Additionally, E-invoicing strengthens supplier and customer relationships by providing a seamless and efficient invoicing approach. This results in accelerated payment cycles, improved customer satisfaction, and decreased costs. Overall, E-invoicing presents substantial advantages for businesses aiming to maintain a competitive edge in today's market [4-11].

In the context of Egypt's mandatory E-invoicing system, research has highlighted the private sector's perceptions of this initiative, focusing on dimensions such as performance expectancy, effort expectancy, social influence, and facilitating conditions. Findings indicate that social influence and facilitating conditions significantly shape perceptions of the E-invoicing system. The acceptance of this system is positively correlated with the size and financial resources of enterprises [12-17]. Nevertheless, concerns persist regarding user control, time-saving capabilities, technical errors, limited coverage, and a lack of digital culture, as well as resistance to change, inadequate infrastructure, and insufficient government communication. Despite these challenges, research offers policy recommendations aimed at enhancing the quality of services associated with the E-invoicing system.

Moreover, the impact of technology adoption and the implementation of electronic data interchange (EDI) and E-invoicing on buyer-seller relationships in the business-to-business market is noteworthy. The research seeks to determine whether the impact varies based on a company's status as an EDI user or a non-EDI company adopting digitalization. Utilizing a theoretical framework and empirical research methods, including qualitative research on three case companies across various sectors, the results demonstrate that the implementation of electronic invoicing influences numerous aspects of business-to-business relationships, with varying degrees of impact. This transformation is often driven by supportive functions, such as finance or accounting [10-15].

## Conclusions

The analysis highlights both challenges and opportunities in the adoption and use of the E-invoice system among medical insurance physicians at Ministry of Health Hospitals. While the system is frequently utilized, significant areas for improvement remain, particularly in addressing technical issues, providing adequate training, and enhancing overall system functionality. Developing tailored training modules is essential to equip physicians with the necessary skills and knowledge to effectively navigate the E-invoice system. Conducting hands-on workshops and offering ongoing support will further empower physicians, allowing them to leverage the system efficiently and ultimately improve workflow efficiency and patient care.

Additionally, addressing the technical challenges faced by physicians is crucial for ensuring smooth and efficient system utilization. Collaboration with IT professionals to enhance system performance and reliability will minimize disruptions and improve user experience. Organizations must prioritize the integration of the E-invoice system with existing hospital workflows to facilitate seamless data exchange and process automation. By monitoring and evaluating system performance, actively soliciting user feedback, and implementing necessary enhancements, organizations can optimize the E-invoice system's functionality, ensuring it meets the evolving needs of healthcare providers and contributes to high-quality patient care.
